# Longitudinal Evaluation of Polyneuropathy in Atypical Parkinsonian Syndromes

**DOI:** 10.3390/neurolint18020027

**Published:** 2026-02-03

**Authors:** Eun Hae Kwon, Julia Steininger, Antonia Bieber, Saskia Kools, Teresa Kleinz, Lovis Hilker, Lea Ebner, Louisa Ortmann, Louisa Basner, Christiane Schneider-Gold, Ralf Gold, Raphael Scherbaum, Kalliopi Pitarokoili, Lars Tönges

**Affiliations:** 1 Department of Neurology, St. Josef-Hospital, Ruhr-University, D-44791 Bochum, Germany; 2 Neurodegeneration Research, Centre for Protein Diagnostics (ProDi), Ruhr-University, D-44791 Bochum, Germany

**Keywords:** polyneuropathy, longitudinal, atypical parkinsonian syndromes, multiple system atrophy, progressive supranuclear palsy, nerve conduction study

## Abstract

**Background:** In Parkinson’s disease (PD), a higher prevalence of polyneuropathy (PNP) is increasingly recognized, although the causal association is still under debate. In contrast, PNP in atypical parkinsonian syndromes (APS) has been insufficiently addressed, despite preliminary evidence suggesting elevated prevalence. **Methods:** Nerve conduction studies were performed on 13 patients with multiple system atrophy (MSA) and 9 patients with progressive supranuclear palsy (PSP) at baseline. PNP was diagnosed according to standard electrophysiological criteria after exclusion of common secondary causes. Comprehensive clinical evaluation included motor and non-motor assessments over two years of follow-up. **Results:** At baseline, PNP was present in 53.8% of MSA patients and 66.7% of PSP patients. MSA patients with PNP showed greater motor symptom severity (UPDRS III score; *p* = 0.046) and worse cognitive performance (MoCA; *p* = 0.044) compared to those without PNP. Over two years, a significant reduction in the tibial nerve amplitude was observed exclusively in MSA patients (*p* = 0.039), paralleling disease progression. **Conclusions:** This study provides the first longitudinal evaluation of clinical and electrophysiological PNP progression in MSA and PSP. A high comorbidity of PNP in patients with APS could contribute to motor and sensory impairments in these patients. Our findings indicate that PNP progression may reflect disease progression in MSA. Given the limited sample size, larger-scale longitudinal studies are needed to further investigate biomarker potential of PNP in APS and to clarify differences in peripheral nerve involvement between synucleinopathies and tauopathies.

## 1. Introduction

Multiple system atrophy (MSA) and progressive supranuclear palsy (PSP) are classified as atypical Parkinsonian syndromes (APS) with clinical and neuropathologic distinctions from Parkinson’s disease (PD). Clinically, MSA presents with parkinsonism, cerebellar ataxia, and early autonomic failure [1]. PSP hallmark features comprise ocular motor dysfunction, akinesia, postural instability, and cognitive impairments, including speech and language disorders [2]. Neuropathologically, MSA is characterized by alpha-synuclein aggregates in oligodendrocytes distributed in olivopontocerebellar and striatonigral structures [1]. PSP represents a 4-repeat-tauopathy predominantly affecting the basal ganglia and brainstem [3].

In recent years, increasing evidence has linked PD to peripheral nervous system involvement. Polyneuropathy (PNP) was found to be more prevalent in PD than in age-matched controls, potentially related to chronic levodopa exposure and interference with vitamin B12 metabolism [4]. Moreover, it is hypothesized that PNP may represent an intrinsic feature of PD as suggested by the detection of alpha-synuclein pathology in peripheral nerve structures [5].

In contrast to the growing body of literature on PD-associated PNP, the relevance of PNP in APS has not been sufficiently addressed yet. In MSA patients, the prevalence of PNP varies from 17.5% to 50% among studies probably due to small study population and heterogeneous application of electrophysiological PNP criteria [6,7,8,9,10]. Even fewer studies have investigated PNP in PSP. Reported prevalences range from approximately 20% up to 65.2% [9,10,11,12]. Importantly, these studies are mainly cross-sectional and provide limited insight into the clinical relevance or temporal evolution of PNP in APS. Our previous cross-sectional “Parkinson Nerve Study”, including patients with MSA and PSP, revealed a high subjective PNP symptom burden [9]. PNP was confirmed electrophysiologically in 50% of patients with both diseases. Notably, compromised motor function correlated with the severity of PNP symptoms in PSP and motor nerve amplitude in MSA. Consequently, PNP comorbidity could aggravate motor and sensory deficits also in APS. To date, longitudinal nerve conduction studies in patients with APS are lacking.

Given this knowledge gap, we extended our “Parkinson Nerve Study” to longitudinally assess the clinical and electrophysiological course of PNP in MSA and PSP patients over a two-year follow-up period. In this study, we investigated whether PNP evolves over time in APS and measures of peripheral nerve function could serve as a surrogate marker of disease progression.

## 2. Materials and Methods

### 2.1. Inclusion of Patients

The cohort of the prospective “Parkinson Nerve Study” was extended at the Department of Neurology of St. Josef-Hospital in Bochum, Germany. This study was approved by the Ethics Committee of the Medical Faculty of Ruhr University Bochum (Reg. No. 18-6360, dated 12 September 2018) and registered in the German clinical trial registry (DRKS-ID: DRKS00020752). All patients provided written informed consent.

Each patient was evaluated by a specialist for movement disorders. Patients fulfilling the diagnostic criteria with at least possible MSA according to the second consensus statement by Gilman et al. (2008) were assessed for eligibility [1]. The PSP criteria established by Höglinger et al. (2017) were used to include PSP patients [2]. The main exclusion criteria were known causes of PNP, such as diabetes mellitus, or history of alcohol abuse.

**Figure 1:**
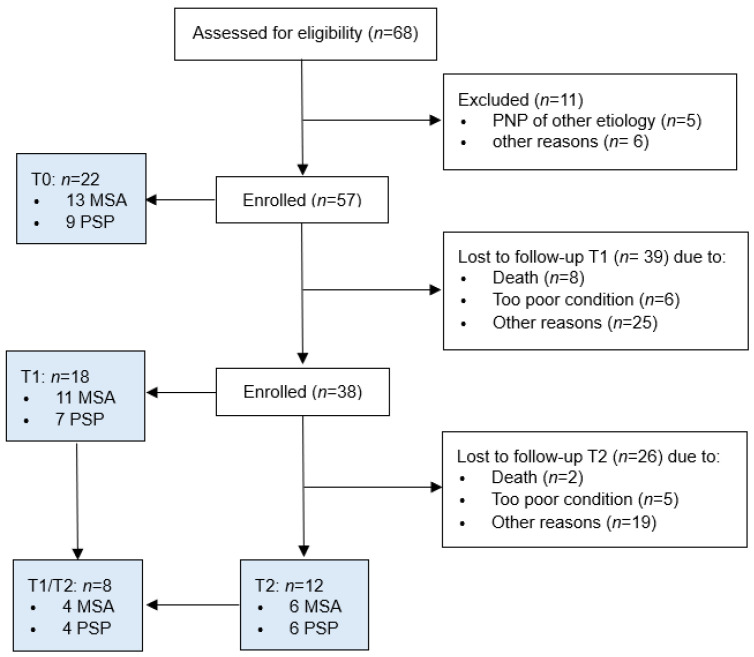
Study flow diagram.

A total of 68 patients were enrolled in the study between 2018 and 2023. Of these, 57 patients were eligible for enrollment. A one-year follow-up examination was conducted on 18 patients (11 MSA, 7 PSP). A two-year follow-up examination was performed on 12 patients (6 MSA, 6 PSP). Reasons for loss of follow-up are presented in Figure 1. A total of 8 patients (4 MSA, 4 PSP) completed follow-up evaluations after a one-year and a two-year interval.

### 2.2. Clinical Evaluation

All patients underwent a physical examination as part of the MDS-Unified Parkinson’s Disease Rating Scale (MDS-UPDRS), part I–III [13]. The disease stage was determined by the modified Hoehn and Yahr Scale [14]. The MSA patients were further evaluated using the Unified MSA Rating Scale (UMSARS) [15] and the PSP patients using the PSP Rating Scale (PSP-RS) [16]. The Neuropathy Symptom Score (NSS) and Neuropathy Deficit Score (NDS) were used to assess PNP [17]. While the NSS focuses on subjective PNP burden, the NDS test assesses clinical PNP parameters. The Montreal Cognitive Assessment (MoCA) was carried out to evaluate memory performance [18]. The patients completed the Parkinson’s Disease Questionnaire—39 (PDQ39) to obtain information on their quality of life [19]. For further assessment of symptoms, they answered the Non-Motor Symptom Questionnaire (NMS) and the Scales for Outcomes in Parkinson’s Disease—Autonomic Dysfunction (SCOPA-AUT) for autonomic nerve issues [20,21].

### 2.3. Assessment of Laboratory Values

Laboratory screening was performed on all patients, comprising blood cell count, HbA1c, liver enzymes, urea, creatinine, electrolytes, thyroid stimulating hormone, vitamin B12, B1, B6, methylmalonic acid, homocysteine, holotranscobalamin levels, and serum protein electrophoresis/immunofixation.

### 2.4. Nerve Conduction Studies

To detect large fiber neuropathy, all patients underwent nerve conduction examinations. Therefore, a Medtronic four-channel electroneurography device (Medtronic, Meerbusch, Germany) was used and classified by the electrophysiological criteria by Stöhr et al., 2014 [22]. The examination contained the study of motor amplitudes of the tibial, fibular, median, and ulnar nerves. The sensory analysis was performed on the fibular, radial, median, and ulnar nerve. Whenever possible, the study was assessed bilaterally. PNP severity was classified into four grades: no, mild, moderate, and severe PNP. The definition of mild neuropathy is based on a reduction of sensory nerve action potential (sNAP) < 3.8 µV in the sural nerve. Moderate neuropathy is defined by an additional reduction of compound muscle action potential (cMAP) < 5 mV in the tibial nerve. Furthermore, if there is a reduction of the median cMAP < 5 mV or a reduced sNAP in the median nerve < 6.9 µV, a severe sensorimotor neuropathy is defined.

### 2.5. Statistical Analysis

All statistical analyses and graphics were performed using SPSS version 29.0.0 (IBM Deutschland GmbH, Ehningen, Germany). Unless otherwise stated, clinical, demographic, and electrophysiological data are shown as means with standard deviations (SD), while ordinal data are shown as median and interquartile range (IQR). Due to the limited sample size, nonparametric tests were applied without prior testing for normality. The Mann–Whitney U test and the Wilcoxon test were applied for group comparison. In addition, bootstrapped *t*-tests were performed for evaluation purposes using 1000 resamples and 95% confidence intervals, with the bias-corrected and accelerated (BCa) method applied by default. Deviations from non-parametric tests are reported in the tables. Logistic regression analyses were conducted for each significant variable. The Friedman test was employed to analyze the data from all three periods. In the post hoc analysis, the Bonferroni–Holm correction was used to control for the type I error due to multiple testing. Differences were considered significant if *p* < 0.05.

## 3. Results

We included 22 patients, 13 diagnosed with MSA and 9 with PSP, for our longitudinal analysis as summarized in Table 1 and Table 2.

Based on nerve conduction studies, 53.8% of our patients with MSA showed signs of at least a mild PNP at baseline. According to our criteria, 15.4% exhibited a mild sensory, 7.7% a moderate sensorimotor, and 30.8% a severe sensorimotor PNP. MSA patients with electrophysiological evidence of PNP were significantly older at examination (*p* = 0.007) and at PD diagnosis (*p* = 0.022) compared to those without PNP (Table 1). Notably, MSA patients with PNP had a significantly lower MoCA result (*p* = 0.044) and a higher UMSARS part III score (*p* = 0.046) (Table 1). Furthermore, NDS, NSS, NMSQ, and PDQ39 did not differ between MSA patients with and without PNP. Interestingly, there were also no differences in the levodopa dose, levodopa equivalence dose (LED), or laboratory assessments. Bootstrapped confidence intervals for the NDS and fibular sensory nerve reached statistical significance at baseline in the MSA cohort. However, given the limited number of cases, these findings should be interpreted with caution. Age-adjusted logistic regression analyses identified significant associations for the sural nerve, tibial nerve, and fibular motor nerve at baseline in the MSA population (Table S1). Owing to the small sample size, these analyses are considered exploratory and hypothesis-generating only.

Overall, 66.7% of our PSP patients showed signs of at least a mild PNP at baseline. In total, 33.3% met the criteria of mild sensory neuropathy, and 33.3% had severe sensorimotor PNP.

In contrast, PSP patients with and without PNP did not differ in age at examination or age at diagnosis (Table 2). No difference could be found in NDS, NSS, NMSQ, PDQ39, and UPDRS I to III. Notably, PSP patients with PNP had a significantly higher MoCA score compared to those without PNP (*p* = 0.027). Consistent with the findings observed in MSA, no discrepancies were detected in levodopa dose, LED, or laboratory. Although bootstrapped confidence intervals for the NSS suggested statistically significant effects, these findings should be interpreted with caution due to the small number of patients. In the PSP cohort at baseline, age-adjusted logistic regression analyses identified a significant association for the sural nerve. Given the limited sample size, these results should be considered exploratory and generalizable only to a limited extent (Table S2).

### 3.1. Follow-Up Evaluation

#### Change of Clinical Data

Both the MDS-UPDRS II (T1: *p* = 0.028; T2: *p* = 0.042) and MDS-UPDRS III (T1: *p* = 0.008; T2: *p* = 0.043) worsened significantly within the one-year and two-year follow-up (Table S3 and Table 3) in the total MSA population. Over the course of one year, we observed a significant deterioration of the PDQ39 (*p* = 0.018), NDS (*p* = 0.045), and UMSARS I (*p* = 0.042) (Table S3). The two-year evaluation revealed a significant deterioration in NMSQ (*p* = 0.039) and MoCA (*p* = 0.039) (Table 3). Subgroup analysis of PNP-positive MSA patients showed no significant change in clinical parameters. In addition to the nonparametric results, bootstrapping suggested significant confidence intervals for NDS and UMSARS I in the overall MSA population at T0–T2 (Table S5). In MSA patients with PNP, positive confidence intervals were also observed for several clinical, biochemical, and neurophysiological measures (Table S6). Given the limited sample size, these findings are exploratory and hypothesis-generating only and require confirmation in larger cohorts.

Over the course of one year, we observed a significant worsening of disease severity according to Hoehn and Yahr stage in the total PSP population (*p* = 0.014) (Table S4). This change could not be maintained at the two-year follow-up evaluation (Table 4). At the two-year follow-up examination, a significant decrease in the MoCA (*p* = 0.043) was found in all PSP patients (Table 4). MDS-UPDRS II increased significantly at T2 in both the total and PNP-positive PSP groups. In addition to the significant findings observed in the nonparametric analyses, bootstrapping suggested statistically significant confidence intervals for H&Y, MDS-UPDRS I, NDS, PSP RS I, PSP RS V, levodopa, and the median sensory nerve in the overall PSP population; however, given the limited sample size, these findings should be interpreted cautiously and are considered exploratory (Table S7).

### 3.2. Change of PNP Parameters

**Figure 2:**
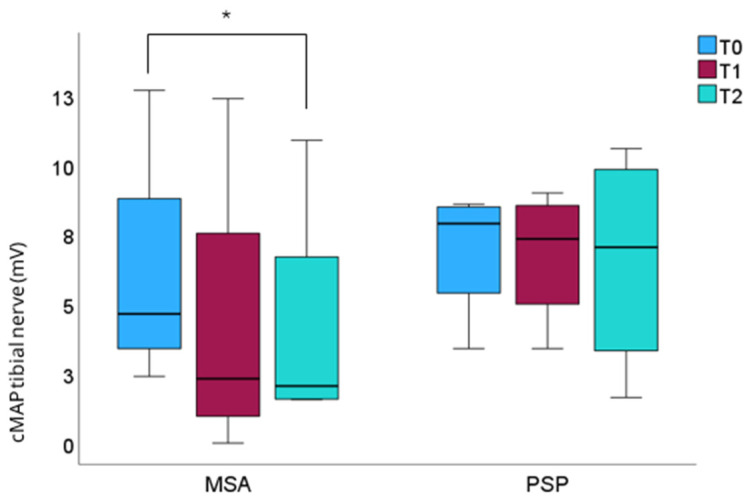
Evolution of the tibial nerve amplitude. Evolution of the tibial nerve amplitude (mV) in the MSA and PSP cohort from baseline to one-year (T1) and two-year (T2) follow-up. * *p*  <  0.05.

While no significant differences in nerve amplitudes were detected after one year of follow-up, the tibial cMAP decreased at the two-year follow-up examination (*p* = 0.028) in the total MSA cohort (Table 3). The tibial cMAP showed a decreasing trend in the group comparison of follow-up examinations (*p* = 0.039) (Figure 2; Table S8).

Regarding the evolution of PNP in MSA patients at the T0–T1 cohort (11 patients), at baseline, six patients showed no electrophysiological signs of PNP (Figure S1). One patient exhibited a mild PNP, and four patients met the criteria for a severe PNP. At the one-year follow-up examination, five patients were found to have no indications of PNP, whereas one patient showed mild and another patient displayed electrophysiological signs of a moderate PNP. Three patients presented with severe PNP.

The cohort of T0–T2 (6 patients) showed one patient with mild, one patient with moderate, and one patient with severe neuropathy (Figure S1). At T2, two patients remained without PNP, and PNP status of the other patients deteriorated to severe grade (Figure S2).

A similar trend was shown in four patients who underwent all three examination periods. At baseline, three patients had no electrophysiological signs of PNP, while one patient had severe PNP. In the subsequent one-year follow-up examination, two patients exhibited no PNP, and two patients demonstrated severe PNP. At the two-year follow-up examination, the number of patients with severe PNP increased to three. The remaining patient did not meet our criteria for PNP (Figure S3).

No significant differences in the nerve amplitudes were observed among PSP patients after one and two years of follow-up (Table S9). At T0, three patients showed no electrophysiological signs of PNP, two patients were classified with mild PNP, and the remaining two met the criteria for severe PNP. At T1, two patients had no PNP, three patients showed signs of mild PNP, and one patient exhibited moderate PNP (Figure S1).

An analysis of the distribution of the six PSP patients, who underwent both T0 and T2 examinations, revealed that the prevalence of PNP remained constant at 5 of 6 patients, while the distribution of PNP severity changed. At the following examination, one patient shifted from mild to moderate PNP (Figure S2).

The four patients who attended all examination periods exhibited a nearly constant distribution of PNP severity. Of these patients, one exhibited no PNP, two patients demonstrated mild PNP, and one patient displayed severe PNP at both the first and last examinations (Figure S3).

## 4. Discussion

This is the first longitudinal study systematically evaluating clinical and electrophysiological progression of PNP in both MSA and PSP patients over the course of two years in order to evaluate peripheral nerve involvement in APS.

PNP was detected by nerve conduction studies in 7 out of 13 MSA patients (53.8%) and 6 out of 9 PSP patients (66.7%) at baseline. PNP was found to be more prevalent in our study compared to other findings. Pramsteller et al. showed mixed sensorimotor axonal neuropathy in 17.5% of 40 MSA patients [6]. Another study reported a PNP rate of 24% in 42 MSA patients with more pronounced axonal loss in the MSA-P than in the MSA-C subgroup [7]. Gawel et al. demonstrated abnormal nerve conduction of one nerve in 20.8% and in two nerves in another 20.8% of a total of 48 MSA patients [8]. As opposed to the assumption of a common length-dependent neuropathy, the ulnar nerve was found to be the most frequently and significantly affected nerve. The same study group performed nerve conduction studies also in 24 PSP patients and found that only the ulnar nerve showed decreased motor and sensory nerve amplitudes in 8.3% and 20% of PSP patients, not different from the control group [12]. Despite few ENG alterations, EMG abnormalities were present in nearly half of the PSP patients, pointing towards a primary lower motoneuron involvement. Another study recorded a higher PNP prevalence in PSP patients (65.2%), in line with our findings, compared to 39.8% in PD patients [11]. The authors concluded a greater affection of myelinated nerve fibers in PSP and more involvement of small fiber nerves in PD. Comparing 104 PD, 52 PSP, and 27 MSA patients, a recent study found no significant difference among all three disease groups, with PNP prevalences ranging between 37.0% and 47.1% [10].

These varying ranges of PNP frequencies could be explained by heterogeneous study populations, and particularly by the application of divergent diagnostic criteria for PNP. In our study, the lower value of bilateral nerve measurement was selected to capture the most clinically relevant degree of peripheral nerve involvement and to ensure consistency throughout the electrophysiological measurements.

In our MSA cohort, patients presenting with PNP were of older age at the time of examination and diagnosis. Age is generally considered a significant risk factor for PNP, with prevalence increasing in older adults due to nerve degeneration and a higher likelihood of related conditions like diabetes [23]. In PD patients, age has also been found to be independently associated with PNP, with increase of PNP risk by 8% for each year of age [24]. PNP also correlated with older age and a more advanced stage of PD, although PNP rate in PD was higher than in age-matched controls [25]. Our previous work ruled out an age-dependent effect after age correction in MSA patients [9]. Furthermore, no differences in terms of age were found between PSP patients with and without PNP.

MSA patients with PNP displayed worse baseline MoCA scores than MSA patients without PNP. In our previous longitudinal analysis of PNP progression in PD, a decrease in sural nerve amplitude correlated with non-motor scores at baseline (PDQ-39 and MoCA) [26]. Cognitive decline is increasingly recognized as a non-motor feature in MSA [27]. It can be speculated that, analogous to PD, presence of PNP could reflect the widespread neurodegenerative changes also in MSA, as the other representative of synucleinopathies. Unlike the MSA group, the PNP-positive PSP patients showed higher MoCA scores. Possible explanations of this unexpected finding could result from selection bias, small sample size effects, and statistical fluctuations. Moreover, PSP encompasses a broad spectrum of clinical phenotypes, including a heterogeneous range of cognitive deficits, which, in addition to the low number of patients, could have contributed to the finding of the better cognitive condition in PNP-positive PSP patients.

Over the course of two years of follow-up, a deterioration in PNP severity could be tracked in the MSA and PSP group. Longitudinal nerve conduction studies in PD are scarce and lacking for APS. A prospective study of PD patients treated with levodopa/carbidopa intestinal gel infusion observed an accelerated two-year incidence of PNP, since high-dosage long-term levodopa medication has been discussed to be causally linked to PD-related PNP [28]. In our longitudinal evaluation of PNP in PD, 21.95% of the patients showed a PNP progression, 78.05% of the patients were stable, and 26.83% remained without PNP [26]. The strongest amplitude reduction was reported in the median sensory nerve (45%) followed by the tibial motor nerve (14.2%), stressing the importance of lower as well as upper nerve involvement in PD for monitoring PNP progression. In this study, the tibial nerve amplitude in the MSA cohort was significantly reduced within two years of follow-up, paralleling motor and non-motor progression. Our previous studies demonstrated an inverse association of the tibial nerve amplitude with more advanced motor stages in PD depicted by Hoehn and Yahr stage and UPDRS part III [26,29]. Therefore, the question arises whether PNP progression could serve as a surrogate marker of MSA progression. Merola et al. found that PNP-positive PD patients were independently associated with worse cognitive, axial motor, autonomic, and other non-motor symptoms and suggested PNP as a peripheral marker of a severe PD phenotype [30]. In contrast, no significant decrease in nerve amplitude could be detected in the PSP cohort. However, a cross-sectional study comparing PSP versus PD reported that PSP patients with PNP had a shorter duration of disease and levodopa use as well as more comprised motor function than PD patients with PNP [11]. No significant PNP progression was found on the individual nerve level for PSP. However, the high prevalence of PNP could contribute to the central mechanisms of postural instability and spinal sensory pathway dysfunction, also in PSP [31,32].

Regarding etiology, extrinsic factors such as levodopa-induced neuropathy and intrinsic features of alpha-synuclein deposition in peripheral nerve structures have been discussed for PD [33]. Long-term levodopa exposure is hypothesized to increase PNP risk by depletion of methylation cofactors, such as vitamin B12, and accumulation of homocysteine, which can alter peripheral nerve homeostasis. In our MSA and PSP cohort, no differences in levodopa dosage or vitamin B12 metabolite status were found.

Neuropathologic evidence of peripheral nerve involvement has been demonstrated in MSA and PSP. Sural nerve biopsy showed a 23% decrease in the density of unmyelinated nerve fibers in MSA [34]. In skin biopsies of MSA patients, aggregates of phosphorylated α-synuclein were detected in unmyelinated somatosensory and autonomic fibers [35,36]. MSA patients showed greater phosphorylated α-synuclein deposition and more widespread peripheral distribution than PD patients, pointing towards a greater multisystemic disease nature [36]. Schwann cells have been discussed to be involved in the pathophysiologic process since α-synuclein accumulation was located in these cell types [37,38,39]. However, the origin of α-synuclein and the exact mechanisms leading to peripheral nerve dysfunction are not sufficiently understood. As for PSP, fewer neuropathologic studies have been performed. Tanaka et al. provided evidence of tau accumulation in cranial and spinal nerves as well as anterior roots to a greater extent in PSP compared to other tauopathies [40]. Interestingly, the study demonstrated tau seeding capacity also in the peripheral nerves by using a tau biosensor assay. Small fiber pathology was also evidenced in PSP patients [41]. Loss of sensory and autonomic nerve fibers was more pronounced in PSP compared to PD and correlated with disease severity. Rong et al. proposed that distinct expression patterns of phosphorylated α-synuclein tau in sural nerve biopsies could help differentiate PD from MSA and PSP, serving as valuable diagnostic biomarkers [42].

The major limitation of our study is the very small sample size, which restricts statistical power and limits the feasibility of conducting more complex or multivariable analyses. As a result, subgroup effects may not have been detected, and the generalizability of the findings should be interpreted with caution. Another important limitation is the high drop-out rate during follow-up evaluations. This attrition was partially attributable to the COVID-19 pandemic, during which social restrictions and disruptions to healthcare access likely discouraged participants from attending scheduled study visits. Such external factors may have disproportionately affected long-term follow-up and data completeness. Moreover, patients with more severe disease may have experienced greater physical and logistic barriers to returning for follow-up assessments. This could have led to bias in the patient composition. Taken together, these limitations highlight the need for cautious interpretation of the results. Future studies with larger sample sizes and improved follow-up strategies may help mitigate these issues and provide more robust and representative findings.

In conclusion, this study represents the first longitudinal analysis of the course of PNP in APS, indicating a high prevalence of PNP in MSA and PSP. In patients with MSA, disease progression was accompanied by a significant decline in tibial nerve amplitudes over time. This finding suggests that peripheral nerve involvement may reflect ongoing neurodegenerative processes. Consequently, PNP may serve as a potential surrogate marker of disease progression in MSA. However, given the very limited sample size, this hypothesis remains exploratory. The observed differences in peripheral nerve involvement highlight potential pathogenic distinctions between synucleinopathies and tauopathies that warrant further investigation. Larger longitudinal studies using standardized nerve conduction protocols and complementary neuropathological assessments are needed to validate these findings and to better understand the clinical relevance of peripheral nerve involvement in APS.

## Figures and Tables

**Table 1:** Clinical and electrophysiological characteristics of the MSA study popualtion with and without PNP at baseline (T0).

	PNP(+) (*n* = 7)	PNP(−) (*n* = 6)	*p*	Lower KI	Higher KI (*Samples*) ^b^
Age at examination (years)	74.14 ± 8.17 (7)	59.67 ± 6.28 (6)	**0.007** **	**−20.4**	**−8.03** (*999*)
Disease duration (years)	2.86 ± 3.13 (7)	2.5 ± 2.17 (6)	0.942	−3.65	2.67
Age at PD diagnosis (years)	71.71 ± 9.51 (7)	57.67 ± 6.77 (6)	**0.022** *	**−22.99**	**−4.53** (*999*)
H&Y (median, IQR)	3 (IQR 1) (7)	2.75 (IQR 1.3) (6)	0.197	−1.2	0.27
MDS-UPDRS I	11.5 ± 3.99 (6)	10.20 ± 4.92 (5)	0.580	−6.16	4.33 (*998*)
MDS-UPDRS II	16.17 ± 7.22 (6)	14.4 ± 7.04 (6)	0.748	−8.98	5.67 (*998*)
MDS-UPDRS III	33.14 ± 13.92 (7)	24.33 ± 12.85 (6)	0.317	−22.81	7.11
UMSARS I	13.5 ± 2.88 (6)	13.4 ± 6.58 (5)	0.581	−5.0	5.27 (*999*)
UMSARS II	20.33 ± 6.83 (6)	17.4 ± 7.83 (5)	0.714	−10.86	5.0 (*999*)
UMSARS III (median, IQR)	1 (IQR 0) (2)	0 (IQR 0) (3)	**0.046** *	-	-
UMSARS IV	2.4 ± 1.14 (5)	2.83 ± 1.04 (3)	0.539	−8.75	2.0 (*979*)
PDQ-39	27.83 ± 10.76 (5)	21.84 ± 14.42 (5)	0.465	−21.85	10.66 (*996*)
NMSQ	10.33 ± 4.27 (6)	7.83 ± 4.07 (6)	0.295	−7.25	3.67 (*999*)
NSS	6.33 ± 3.27 (6)	3.83 ± 3.06 (6)	0.126	−6.17	0.71 (*999*)
NDS	4.17 ± 1.84 (6)	1.67 ± 2.86 (6)	0.102	**−4.7**	**−0.17** (*998*)
MoCA	20.71 ± 3.25 (7)	24.33 ± 2.25 (6)	**0.044** *	**0.652**	**6.612**
LED (mg)	671.81 ± 532.76 (7)	589.17 ±177.83 (6)	0.668	−583.32	275.13 (*998*)
Levodopa (mg)	514.29 ± 295.40 (7)	366.67 ±147.20 (6)	0.561	−393.75	69.64 (*997*)
Vitamin B12 (pg/mL)	415.29 ± 209.28 (7)	627.33 ± 683.81 (6)	1.0	−191.08	864.77 (*999*)
Holotranscobalamin (pmol/L)	94.00 ± 42.32 (7)	92.32 ± 36.89 (6)	0.886	−42.08	36.42 (*999*)
Folic acid (ng/mL)	11.02 ± 7.20 (7)	8.81 ± 4 (6)	0.775	−8.4	3.69 (*999*)
Methylmalonic acid (nmol/L)	339.02 ± 60.71 (6)	321.16 ± 188.60 (5)	0.855	−185.06	138.27 (*995*)
Homocysteine (µmol/L)	18.5 ± 3.05 (6)	17.73 ± 6.52 (6)	0.873	−6.69	5.5 (*999*)
Sural nerve (µV)	0.33 ± 0.57 (7)	6.88 ± 2.16 (6)	**0.002** **	**4.99**	**8.18**
Tibial nerve (mV)	3.61 ± 2.26 (7)	10.5 ± 6.24 (6)	**0.046** *	**1.41**	**12.24** (*997*)
Median motor nerve (mV)	6.13 ± 2.34 (7)	5.6 ± 2.89 (6)	0.668	−3.23	2.58
Median sensory nerve (µV)	6.52 ± 4.93 (6)	5.12 ± 1.5 (6)	0.936	−6.06	2.02 (*998*)
Fibular motor nerve (mV)	1.7 ± 0.99 (4)	6.14 ± 1.95 (6)	0.011 *	2.63	6.29 (*993*)
Fibular sensory nerve (µV)	0 ± 0 (4)	1.25 ± 2.04 (5)	0.180	**0.26 ^a^**	3.13 (*869*)
Radial nerve (µV)	5.38 ± 2.21 (4)	4.47 ± 3.06 (6)	0.593	−4.49	2.64 (*987*)
Ulnar motor nerve (mV)	6.9 ± 1.94(4)	7.28 ± 2.48 (6)	0.914	−2.2	2.95 (*988*)
Ulnar sensory nerve (µV)	5.37 ± 3.6 (4)	6.83 ± 1.16 (6)	0.352	−2.98	4.92 (*990*)

PNP(+) MSA patients with PNP, PNP(−) MSA patients without PNP. IQR interquartile ratio, H&Y Hoehn and Yahr Scale, MDS-UPDRS Movement Disorder Society Unified Parkinson’s Disease Rating Scale, UMSARS Unified Multiple System Atrophy Rating Scale, PDQ-39 Parkinson’s Disease Questionnaire 39, NMSQ Non-Motor Symptom Questionnaire, NSS Neuropathy Symptom Score, NDS Neuropathy Disability Score, MoCA Montreal Cognitive Assessment, LED levodopa equivalence dose, LD levodopa dose. Bold is used for statistically significant results. * *p*  <  0.05; ** *p*  <  0.01. Clinical scores: mean values  ±  standard deviation (SD) are presented. H&Y scale: median value and IQR are presented. Confidence intervals were considered significant if they did not include the value zero. ^a^ If it could not be calculated using the BCa method, then the percentile method was used, ^b^ if not otherwise stated, bootstrapping was based on 1000 samples and indicated in brackets in italics.

**Table 2:** Clinical and electrophysiological characteristics of the PSP study popualtion with and without PNP at baseline (T0).

	PNP(+) (*n* = 6)	PNP(−) (*n* = 3)	*p*	Lower KI	Higher KI (*Samples*) ^b^
Age at examination (years)	70.33 ± 9.77 (6)	71.67 ± 5.03 (3)	0.795	−7.74 ^a^	10.5 (*963*)
Female	2	0	0.073	-	-
Disease duration (years)	3.67 ± 3.98 (6)	1.33 ± 2.31 (3)	0.294	−6.89	2.0 (*962*)
Age at PD diagnosis (years)	66.67 ± 12.79 (6)	70.33 ± 5.77 (3)	0.793	−7.42 ^a^	16.12 (*956*)
H&Y (median, IQR)	3 (IQR 1.3)	3 (IQR 0)	0.773	−0.6 ^a^	1.1 (*952*)
MDS-UPDRS I	10 ± 6.9 (6)	10 ± 2 (3)	1.0	−6.33 ^a^	5.9 (*961*)
MDS-UPDRS II	19.67 ± 12.83 (6)	19.33 ± 5.13 (3)	0.897	−11.22 ^a^	10.79 (*955*)
MDS-UPDRS III	41.83 ± 15.5 (6)	33.67 ± 12.05 (3)	0.606	−32.75	16.42
PSP-RS I–VI	42.67 ± 18.93 (3)	40.67 ± 14.57 (3)	1.0	−20	20 (*957*)
PSP-RS I	6.75 ± 4.99 (4)	8.67 ± 5.03 (3)	0.480	−5	9.5 (*973*)
PSP-RS II	5 ± 3.56 (4)	5.67 ± 0.58 (3)	0.857	−2.1 ^a^	4.31 (*970*)
PSP-RS III	2.5 ± 1 (4)	2.33 ± 3.21 (3)	0.578	−2.67	3.67 (*950*)
PSP-RS IV	6 ± 4.76 (4)	9.33 ± 1.15 (3)	0.476	−0.83	7.48 (*970*)
PSP-RS V	5.2 ± 1.92 (5)	4 ± 1 (3)	0.362	−3.58	0.95 (*973*)
PSP-RS VI	12 ± 4.69 (4)	10.67 ± 4.73 (3)	0.724	−6.65 ^a^	5.17 (*971*)
PDQ-39	28.64 ± 19.38 (5)	19.69 ± 10.61 (2)	0.699	−29.87	15.11 (*873*)
NMSQ	8 ± 4.69 (6)	8.33 ± 1.16 (3)	0.895	−3.33 ^a^	4.21 (*946*)
NSS	3.33 ± 4.27 (6)	0 ± 0 (3)	0.167	**−6.26**	**−0.75** (*926*)
NDS	4.5 ± 1.52 (6)	5.33 ± 1.16 (3)	0.410	−0.88 ^a^	2.5 (*945*)
MoCA	23.5 ± 3.78 (6)	17.33 ± 1.53 (3)	**0.027** *	**−9.63** ^a^	**−2.85** (*952*)
LED (mg)	398.67 ± 240.14 (6)	533.33 ± 550.76 (3)	0.895	−548.1	819.14 (*965*)
Levodopa (mg)	216.67 ± 240.14 (6)	400 ± 400 (3)	0.492	−242.81	619.97 (*938*)
Vitamin B12 (pg/mL)	325.67 ± 131.13 (6)	681.67 ± 458.08 (3)	0.302	−72.64 ^a^	890.5 (*964*)
Holotranscobalamin (pmol/L)	100.35 ± 55.48 (6)	93.3 ± 52.95 (3)	1.0	−76.84 ^a^	65.64 (*944*)
Folic acid (ng/mL)	8.46 ± 5.76 (6)	11.28 ± 7.65 (3)	0.302	−4.35	11.91 (*950*)
Methylmalonic acid (nmol/L)	182.6 ± 131.13 (6)	258.27 ± 207.48 (3)	0.796	−139.15	338.28 (*959*)
Homocysteine (µmol/L)	20.08 ± 7.58 (5)	16.23 ± 3.18 (3)	0.456	−10.39	3.17 (*959*)
Sural nerve (µV)	1.68 ± 1.85 (6)	4.27 ± 0.25 (3)	**0.018** *	**0.92** ^a^	**4.17** (*955*)
Tibial nerve (mV)	5.77 ± 2.03 (6)	7.67 ± 2.06 (3)	0.121	−1.14 ^a^	4.27 (*965*)
Median motor nerve (mV)	5.08 ± 1.73 (6)	4.9 ± 2.08 (3)	0.604	−2.83	2.97 (*954*)
Median sensory nerve (µV)	8.02 ± 4 (5)	4.83 ± 1.45 (3)	0.297	−6.91 ^a^	0.58 (*941*)
Fibular motor nerve (mV)	2.36 ± 2.38 (4)	3.77 ± 2.37 (3)	0.154	−1.93 ^a^	4.91 (*927*)
Fibular sensory nerve (µV)	0.85 ± 1.7 (4)	0 ± 0 (3)	0.386	−2.74 ^a^	0.00 (*671*)
Radial nerve (µV)	6.55 ± 2.53 (4)	5.63 ± 0.81 (3)	1.0	−4.52 ^a^	1.05 (*957*)
Ulnar motor nerve (mV)	6.38 ± 2.66 (4)	6.53 ± 2.28 (3)	0.724	−3.1 ^a^	3.7 (*939*)
Ulnar sensory nerve (µV)	4.78 ± 3.54 (4)	5.6 ± 0.5 (3)	1.0	−2.19	5.35 (*945*)

PNP(+) PSP patients with PNP, PNP(−) PSP patients without PNP. IQR interquartile ratio, H&Y Hoehn and Yahr Scale, MDS-UPDRS Movement Disorder Society Unified Parkinson’s Disease Rating Scale, PSP-RS PSP Rating Scale, PDQ-39 Parkinson’s Disease Questionnaire 39, NMSQ Non-Motor Symptom Questionnaire, NSS Neuropathy Symptom Score, NDS Neuropathy Disability Score, MoCA Montreal Cognitive Assessment, LED levodopa equivalence dose, LD levodopa dose. Bold is used for statistically significant results. * *p*  <  0.05 Clinical scores: mean values  ±  standard deviation (SD) are presented. H&Y scale: median value and IQR are presented. Confidence intervals were considered significant if they did not include the value zero. ^a^ If it could not be calculated using the BCa method, then the percentile method was used, ^b^ if not otherwise stated, bootstrapping was based on 1000 samples and indicated in brackets in italics.

**Table 3:** Longitudinal evaluation of the MSA cohort at T0 and T2.

	T0 Total (*n* = 6)	T2 Total (*n* = 6)	*p*	T0 PNP(+) (*n* = 3)	T2 PNP(+) (*n* = 3)	*p*
H&Y	3 (IQR 0.4) (6)	3.5 (IQR 1.5) (6)	0.285 (6)	3 (IQR 0) (3)	4 (IQR 0) (3)	0.317 (3)
MDS-UPDRS I	9.25 ± 5.19 (4)	14.17 ± 4.75 (6)	0.197 (4)	12 ± 7.01 (2)	15.67 ± 6.66 (3)	0.655 (2)
MDS-UPDRS II	16.00 ± 6.40 (5)	29.33 ± 6.86 (6)	**0.042** * (5)	21 ± 5.66 (2)	33.67 ± 3.51 (3)	0.180 (2)
MDS-UPDRS III	26.67 ± 14.25 (6)	43.8 ± 6.42 (5)	**0.043** * (5)	31.33 ± 16.92 (3)	47 ± 4.36 (3)	0.109 (3)
PDQ 39	19.13 ± 19.47 (3)	44.22 ± 20.98 (4)	0.180 (2)	40.21 (1)	56.49 ± 19.78 (2)	-
NMSQ	6.60 ± 3.44 (5)	11.5 ± 3.02 (6)	**0.039** * (5)	8.5 ± 4.95 (2)	13 ± 3 (3)	0.180 (2)
NSS	4.00 ± 3.81 (5)	4 ± 3.1 (6)	0.854 (5)	7.5 ± 0.71 (2)	4 ± 3.46 (3)	0.180 (2)
NDS	2.4 ± 2.30 (5)	5.33 ± 2.81 (6)	0.066 (5)	4.5 ± 0.71 (2)	7 ± 1 (3)	0.180 (2)
UMSARS I	13.00 ± 4.08 (4)	20.83 ± 5.64 (6)	0.068 (4)	15 ± 1.41 (2)	23.33 ± 4.04 (3)	0.180 (2)
UMSARS II	20.50 ± 9.33 (4)	20.4 ± 4.28 (5)	1.00 (3)	24.5 ± 10.61 (2)	22.33 ± 4.73 (3)	0.655 (2)
UMSARS III	0.00 (1)	0.33 ± 5.8 (3)	1.00 (1)	-	1 (1)	-
UMSARS IV	2.50 ± 0.50 (3)	2.75 ± 1.5 (4)	0.317 (1)	2.5 ± 0.71 (2)	4 (1)	-
MoCA	22.17 ± 3.66 (6)	19.5 ± 4.14 (6)	**0.039** * (6)	19.33 ± 1.53 (3)	17.33 ± 2.08 (3)	0.157 (3)
LED (mg)	523.22 ± 176.24 (6)	718.06 ± 301.31 (6)	0.075 (6)	419.78 ± 149.05 (3)	483.33 ± 101.04 (3)	0.285 (3)
Levodopa (mg)	358.33 ± 91.74 (6)	520 ± 164.3 (5)	0.104 (5)	383.33 ± 125.83 (3)	433.33 ± 152.75 (3)	0.414 (3)
Vitamin B12 (pg/mL)	334.33 ± 121.73 (6)	374.4 ± 94.25 (5)	0.345 (5)	315.67 ± 59.55 (3)	414.5 ± 123.74 (2)	0.180 (2)
Holotranscobalamin (pmol/L)	81.25 ± 41.27 (6)	79.18 ± 12.61 (5)	0.686 (5)	88.13 ± 53.58 (3)	83.7 ± 15.52 (3)	1.0 (3)
Folic acid (ng/mL)	9.78 ± 6.12 (6)	6.43 ± 3.25 (5)	0.715 (5)	10.32 ± 8.67 (3)	7.4 ± 5.52 (2)	0.180 (2)
Methylmalonic acid (nmol/L)	409.50 ± 114.62 (4)	449.3 ± 193.54 (5)	0.285 (3)	372.05 ± 62.15 (2)	495.67 ± 197.68 (3)	0.180 (2)
Homocysteine (µmol/L)	19.98 ± 4.79 (5)	19.36 ± 3.49 (5)	0.273 (4)	19.85 ± 1.91 (2)	20.4 ± 4.21 (3)	0.180 (2)
Sural nerve (µV)	2.8 ± 3.13 (6)	2.93 ± 4.9 (6)	0.854 (6)	0 ± 0 (3)	1.97 ± 3.41 (3)	0.317 (3)
Tibial nerve (mV)	5.7 ± 3.78 (6)	3.88 ± 3.76 (6)	**0.028** * (6)	4.73 ± 2.02 (3)	3.08 ± 2.1 (3)	0.109 (3)
Median sensory nerve (µV)	6.82 ± 4.77 (6)	3.85 ± 2.08 (6)	0.116 (6)	8.57 ± 6.82 (3)	4.8 ± 1.75 (3)	0.285 (3)
Median motor nerve	5.27 ± 2.7 (6)	5.7 ± 3.31 (6)	0.343 (6)	5.67 ± 3.22 (3)	6.43 ± 4.29 (3)	0.285 (3)
Fibular motor nerve (mV)	3.73 ± 2.02 (5)	2.4 ± 1.95 (5)	0.068 (4)	1.75 ± 1.06 (2)	3.22 ± 3.37 (2)	0.317 (1)
Fibular sensory nerve (µV)	0.00 ± 0.00 (5)	0.71 ± 1.04 (5)	0.317 (4)	0 ± 0 (2)	1.15 ± 1.63 (2)	1.0 (1)
Radial nerve (µV)	4.88 ± 2.06 (5)	5.4 ± 2.14 (4)	0.285 (3)	5.55 ± 3.75 (2)	4.1 ± 0.71 (2)	0.317 (1)
Ulnar motor nerve (mV)	7.04 ± 1.7 (5)	8.52 ± 1.21 (5)	0.144 (4)	8.05 ± 2.05 (2)	9.3 ± 1.7 (2)	0.317 (1)
Ulnar sensory nerve (µV)	7.02 ± 1.89 (5)	2.86 ± 2.99 (5)	0.068 (4)	8.15 ± 2.62 (2)	4.15 ± 2.47 (2)	0.317 (1)

* Clinical scores: mean values  ±  SD are presented. H&Y scale: median value and IQR are presented. NCS: mean amplitudes  ±  SD are presented. H&Y: median and IQR are presented. Bold is used for statistically significant results. * *p*  <  0.05.

**Table 4:** Longitudinal evaluation of the PSP cohort at T0 and T2.

	T0 Total (*n* = 6)	T2 Total (*n* = 6)	*p*	T0 PNP(+) (*n* = 5)	T2 PNP(+) (*n* = 5)	*p*
H&Y	3 (IQR 0.5) (6)	3 (IQR 1.3) (6)	0.317 (6)	3 (IQR 1) (5)	3 (IQR 1.5) (5)	0.317 (5)
MDS-UPDRS I	11.00 ± 6.63 (6)	14.33 ± 5.57 (6)	0.080 (6)	10.8 ± 7.4 (5)	13.4 ± 5.68 (5)	0.144 (5)
MDS-UPDRS II	17.00 ± 10.75 (6)	25.67 ± 12.44 (6)	**0.027** * (6)	16.8 ± 12.01 (5)	26.8 ± 13.55 (5)	**0.042** * (5)
MDS-UPDRS III	34.00 ± 10.79 (6)	41.83 ± 15 (6)	0.249 (6)	36.6 ± 9.74 (5)	42.8 ± 16.55 (5)	0.5 (5)
PDQ 39	24.79 ± 20.05 (4)	42.17 ± 23.98 (5)	0.593 (3)	24.79 ± 20.05 (4)	43.89 ± 27.33 (4)	0.593 (3)
NMSQ	8.00 ± 4.69 (6)	10.33 ± 5.75 (6)	0.172 (6)	7.8 ± 5.22 (5)	10 ± 6.36 (5)	0.223 (5)
NSS	3.33 ± 4.27 (6)	4.33 ± 4.13 (6)	0.715 (6)	4 ± 4.42 (5)	5.2 ± 3.96 (5)	0.715 (5)
NDS	4.50 ± 1.52 (6)	5.67 ± 2.58 (6)	0.180 (6)	4.2 ± 1.48 (5)	5.6 ± 2.88 (5)	0.180 (5)
PSP RS I–VI	33 ± 15.87 (3)	31.67 ± 13.87 (6)	1.0 (3)	36 ± 21.21 (2)	32.4 ± 15.37 (5)	0.655 (2)
PSP RS I	5.25 ± 4.57 (4)	7± 4.2 (6)	0.066 (4)	5.67 ± 5.51 (3)	6.8 ± 4.66 (5)	0.109 (3)
PSP RS II	4.50 ± 3.32 (4)	2.33 ± 1.51 (6)	0.141 (4)	4.33 ± 4.04 (3)	2.2 ±1.64 (5)	0.285 (3)
PSP RS III	1.75 ± 1.50 (4)	1.67 ± 2.25 (6)	0.414 (4)	2.33 ± 1.15 (3)	2 ± 2.35 (5)	0.414 (3)
PSP RS IV	5.25 ± 3.86 (4)	6.33 ± 2.5 (6)	0.581 (4)	4.33± 1.16 (3)	6.6 ± 2.7 (5)	0.414 (3)
PSP RS V	4.20 ± 1.30 (5)	4.67 ± 1.97 (6)	0.180 (5)	4.5 ± 1.29 (4)	5 ± 2 (5)	0.180 (4)
PSP RS VI	9.50 ± 3.70 (4)	9.5 ± 4.76 (6)	0.357 (4)	20.33 ± 4.04 (3)	9.6 ± 5.32 (5)	0.593 (3)
MoCA	23.50 ± 3.78 (6)	20 ± 2.97 (6)	**0.043** * (6)	24.4 ± 3.44 (5)	21 ± 1.87 (5)	0.068 (5)
LED (mg)	315.33 ± 365.43 (6)	473.33 ± 448.21 (6)	0.138 (6)	378.4 ± 370.26 (5)	468 ± 500.91 (5)	0.273 (5)
Levodopa (mg)	133.33 ± 206. 56 (6)	275 ± 331.29 (6)	0.102 (6)	160 ± 219.09 (5)	250 ± 364.01 (5)	0.180 (5)
Vitamin B12 (pg/mL)	321.83 ± 171.86 (6)	416.17 ± 129.53 (6)	0.917 (6)	327 ± 191.62 (5)	457 ± 92.02 (5)	0.686 (5)
Holotranscobalamin (pmol/L)	98.62 ± 57.29 (6)	75.82 ± 31.3 (6)	0.600 (6)	109.28 ± 57 (5)	81.68 ± 31.09 (5)	0.5 (5)
Folic acid (ng/mL)	8.22 ± 5.82 (6)	8.72 ± 6.82 (6)	0.500 (6)	8.58 ± 6.43 (5)	9.59 ± 7.24 (5)	0.715 (5)
Methylmalonic acid (nmol/L)	213.40 ± 177.50 (6)	235.57 ± 38.27 (6)	0.753 (6)	159.28 ± 131.96 (5)	244.04 ± 35.95 (5)	0.225 (5)
Homocysteine (µmol/L)	19.18 ± 7.41 (5)	17.07 ± 4.56 (6)	0.138 (5)	19.35 ± 8.55 (4)	17.14 ± 5.09 (5)	0.273 (4)
Sural nerve (µV)	1.83 ± 2.02 (6)	2.58 ± 3.28 (6)	1.0 (6)	1.4 ± 1.92 (5)	2.36 ± 3.62 (5)	0.655 (5)
Tibial nerve (mV)	6.57 ± 2.05 (6)	5.2 ± 3.94 (6)	0.173 (6)	6.16 ± 2 (5)	4.12 ± 3.26 (5)	0.080 (5)
Median sensory nerve (µV)	8.18 ± 3.76 (5)	6.22 ± 1.88 (6)	0.138 (5)	9.25 ± 3.35 (4)	6.7 ± 1.63 (5)	0.144 (4)
Median motor nerve	5.07 ± 1.75 (6)	5.4 ± 2.43 (6)	0.917 (6)	5.34 ± 1.81 (5)	5.88 ± 2.38 (5)	0.686 (5)
Fibular motor nerve (mV)	2.91 ± 1.92 (4)	2.24 ± 2.62 (6)	0.715 (4)	3.08 ± 2.31 (3)	1.47 ± 2.02 (5)	0.109 (3)
Fibular sensory nerve (µV)	0.85 ± 1.7 (4)	0 ± 0 (6)	0.317 (4)	1.13 ± 1.96 (3)	0 ± 0 (5)	0.317 (3)
Radial nerve (µV)	6.88 ± 2.32 (4)	5.6 ± 2.42 (5)	0.593 (3)	7.1 ± 2.79 (3)	5.6 ± 2.42 (5)	0.593 (3)
Ulnar motor nerve (mV)	7.63 ± 1.18 (4)	7.9 ± 2.42 (5)	0.180 (3)	7.57 ± 1.44 (3)	7.9 ± 2.41 (5)	0.180 (3)
Ulnar sensory nerve (µV)	6.3 ± 1.56 (4)	6.7 ± 2.86 (5)	0.593 (3)	6.37 ± 1.91 (3)	6.7 ± 2.86 (5)	0.593 (3)

Clinical scores: mean values  ±  SD are presented. H&Y scale: median value and IQR are presented. NCS: mean amplitudes  ±  SD are presented. H&Y: median and IQR are presented. Bold is used for statistically significant results. * *p*  <  0.05.

## Data Availability

The data are not publicly available due to the privacy concern raised by our IRB.

## Supplementary Materials

The following supporting information can be downloaded at: https://www.mdpi.com/article/10.3390/neurolint18020027/s1. Table S1. Regression analyses for significant values in the MSA cohort at baseline. Table S2. Regression analyses for significant values in the PSP cohort at baseline. Table S3. Longitudinal evaluation of the MSA cohort at T0 and T1. Table S4. Longitudinal evaluation of the PSP cohort at T0 and T1. Table S5. Differences between bootstrapping and nonparametric tests MSA T0-T2 cohort. Table S6. Differences between bootstrapping and nonparametric tests MSA T0–T2 PNP positive cohort. Table S7. Differences between bootstrapping and nonparametric tests PSP T0–T2 cohort. Table S8. Longitudinal evaluation of the MSA cohort at T0, T1 and T2. Table S9. Longitudinal evaluation of the PSP cohort at T0, T1 and T2. Figure S1. (a) Distribution of PNP severity levels in MSA and PSP patients: (a) at baseline; (b) at one.-year follow-up (T1). Figure S2. (a) Distribution of PNP severity levels in MSA and PSP patients: (a) at baseline; (b) at one-year follow-up (T1). Figure S3. (a) Distribution of PNP severity levels of MSA and PSP patients: (a) at baseline; (b) at one-year follow-up (T1); (c) at two year follow-up (T2).

## Abbreviations

The following abbreviations are used in this manuscript:

PD	Parkinson’s disease
APS	Atypical Parkinsonian syndromes
MSA	Multiple system atrophy
PSP	Progressive supranuclear palsy
PNP	Polyneuropathy
sNAP	Sensory nerve action potential
cMAP	Compound muscle action potential
