# Supplementary material for: Longitudinal Evaluation of Polyneuropathy in Atypical Parkinsonian Syndromes
Source: Neurol Int. 2026 Feb 3;18(2):27. doi: 10.3390/neurolint18020027 (PMC12943340; doi:10.3390/neurolint18020027)

Supplementary materials

Table S1. Regression analyses for significant values in the MSA cohort at baseline.

	**Regression coefficient b**	**Exp(b)**	**p** *(samples)*[a]
MoCA	-1.225	0.294	0.058 *(858)*
UMSARS III	-	-	-
Sural nerve (μV)	-10.471	0.00	**0.006** *(179)*
Tibial nerve (mV)	-0.689	0.502	**0.011** *(844)*
Fibular motor nerve (mV)	-15.295	0.00	**0.048** *(392)*
Fibular sensory nerve (μV)	-11.864	0.00	0.221 *(543)*
NDS	0.31	1.363	0.127 *(820)*

[a] if not otherwise stated, bootstrapping was based on 1000 samples.

Table S2. Regression analyses for significant values in the PSP cohort at baseline.

	**Regression coefficient b**	**Exp (b)**	**p** *(samples)*[a]
MoCA	52.001	3.834E+22	0.215 *(566)*
NSS	7.275	1444.196	0.237 *(815)*
Sural nerve (μV)	-33.548	0.00	**0.006** *(471)*

[a] if not otherwise stated, bootstrapping was based on 1000 samples.

Table S3. Longitudinal evaluation of the MSA cohort at T0 and T1.

	**T0 total (n=11)**	**T1 total (n=11)**	**p**	**T0 PNP(+) (n=5)**	**T1 PNP(+) (n=5)**	**p**
**H&Y**	3 (IQR 0,5) (11)	3 (IQR 1,5) (11)	0.083 (11)	3 (IQR 1,5) (5)	4 (IQR 1,3) (5)	0.157 (5)
**MDS-UPDRS I**	10.67 ± 4 (9)	14.63 ± 7.91 (8)	0.235 (7)	11.25 ± 3.1 (4)	13 ± 8.66 (3)	0.593 (3)
**MDS-UPDRS II**	14.20 ± 6.73 (10)	20.88 ± 4.91 (8)	**0.028*** (7)	13.75 ± 7.27 (4)	23 ± 5.72 (4)	0.109 (3)
**MDS-UPDRS III**	30.36 ± 14.39 (11)	42.5 ± 18.24 (10)	**0.008**** (10)	37.60 ± 13.81 (5)	49.75 ± 28.27 (4)	0.068 (4)
**PDQ 39**	23.13 ± 11.87 (9)	39.44 ± 21.31 (9)	**0.018*** (7)	24.74 ± 9.52 (4)	44.9 ± 18.55 (4)	0.109 (3)
**NMSQ**	9.20 ± 4.32 (10)	11.3 ± 5.5 (10)	0.406 (9)	11.25 ± 4.35 (4)	12 ± 3.54 (5)	1.00 (4)
**NSS**	4.91 ± 3.39 (11)	5 ± 2.97 (11)	0.964 (11)	6.2 ± 3.63 (5)	5.2 ± 3.19 (5)	0.498 (5)
**NDS**	2.82 ± 2.75 (11)	4.82 ± 2.18 (11)	**0.045*** (11)	4.2 ± 2.05 (5)	6.6 ± 1.67 (5)	0.104 (5)
**UMSARS I**	13.11 ± 5.09 (9)	16 ± 4.69 (10)	**0.042*** (9)	12.75 ± 3.3 (4)	13.75 ± 1.89 (4)	0.581 (4)
**UMSARS II**	17.78 ± 6.28 (9)	20.64 ± 7.49 (11)	0.810 (9)	18.25 ± 4.79 (4)	22.4 ± 9.34 (5)	0.461 (4)
**UMSARS III**	0.40 ± 0.55 (5)	0.33 ± 0.52 (6)	1.00 (3)	1.00 ± 0 (2)	0 ± 0 (3)	0.317 (1)
**UMSARS IV**	2.58 ± 1.20 (6)	2.89 ± 0.93 (9)	0.655 (5)	2.33 ± 1.53 (3)	3 ± 1.15 (4)	0.317 (3)
**MoCA**	23.09 ± 3.08 (11)	22.09 ± 2.98 (11)	0.282 (11)	21.6 ± 3.51 (5)	20.20 ± 2.59 (5)	0.197 (5)

**LED (mg)**	657.21 ± 429.16 (11)	819.58 ± 450.51 (11)	0.066 (11)	738.87 ± 636.9 (5)	840 ± 661.11 (5)	0.593 (5)
**Levodopa (mg)**	445.45 ± 264.06 (11)	547.73 ± 332.5 (11)	0.051 (11)	540 ± 356.02 (5)	630 ± 468.51 (5)	0.285 (5)
**Vitamin B12 (pg/ml)**	549.91 ± 514.05 (11)	471.67 ± 189.47 (9)	0.314 (9)	457 ± 237.37 (5)	528.75 ± 169.43 (4)	0.715 (4)
**Holotranscobalamin (pmol/l)**	91.36 ± 36.03 (11)	97.11 ± 29.62 (8)	0.161 (8)	90.22 ± 39.26 (5)	106.07 ± 28.47 (3)	0.593 (3)
**Folic acid (ng/ml)**	10.82 ± 5.90 (11)	12.17 ± 6.58 (9)	0.05 (9)	13.24 ± 7.33 (5)	12.55 ± 8.61 (4)	0.593 (4)
**Methylmalonic acid (nmol/l)**	322.9 ± 130.65 (10)	317.82 ± 111.84 (9)	0.889 (8)	323.62 ± 53.19 (5)	404.81 ± 90.46 (4)	0.273 (4)
**Homocysteine (µmol/l)**	17.84 ± 5.00 (11)	17.78 ± 3.54 (9)	0.635 (9)	17.96 ± 3.07 (5)	20.45 ± 3 (4)	0.068 (4)
**Sural nerve (µV)**	3.96 ± 3.71 (11)	2.95 ± 3.28 (10)	0.327 (10)	0.46 ± 0.64 (5)	3.05 ± 3.82 (4)	0.285 (4)
**Tibial nerve (mV)**	7.14 ± 6.02 (11)	4.47 ±5.17 (11)	0.050 (11)	3.1 ± 2.13 (5)	2.67 ± 1.35 (5)	0.686 (5)
**Median sensory nerve (µV)**	4.67 ± 1.48 (10)	3.55 ± 3.4 (11)	0.444 (10)	4 ± 1.36 (4)	4.59 ± 3.27 (5)	0.465 (4)
**Median motor nerve**	5.85 ± 2.36 (11)	6.65 ± 2.83 (11)	0.563 (11)	6.16 ± 1.8 (5)	6.4 ± 1.47 (5)	1.0 (5)
**Fibular motor nerve (mV)**	4.57 ± 2.86 (9)	2.08 ± 1.11 (9)	0.091 (7)	1.44 ± 1.02 (3)	2.08 ± 0.89 (5)	0.593 (3)
**Fibular sensory nerve (µV)**	0.78 ± 1.67 (8)	1.88 ± 3.68 (8)	0.285 (6)	0 ± 0 (3)	1.3 ± 2.6 (4)	0.317 (3)
**Radial nerve (µV)**	4.46 ± 2.53 (9)	5.23 ± 3.38 (10)	0.263 (8)	4.43 ± 1.42 (3)	4.32 ± 3.26 (5)	0.593 (3)
**Ulnar motor nerve (mV)**	6.87 ± 2.13 (9)	7.84 ± 1.64 (10)	0.123 (8)	6.03 ± 1.07 (3)	8.48 ± 1.89 (5)	0.109 (3)
**Ulnar sensory nerve (µV)**	5.83 ± 2.09 (9)	6.23 ± 6.82 (10)	1.0 (8)	3.82 ± 2.26 (3)	5.78 ± 6.75 (5)	1.0 (3)

* Clinical scores: mean values ± SD are presented. H&Y scale: median value and IQR are presented. NCS: mean amplitudes ± SD are presented. H&Y: median and IQR are presented. *$p < 0.05$, **$p < 0.01$.

Table S4. Longitudinal evaluation of the PSP cohort at T0 and T1

	**T0 total (n=7)**	**T1 total (n=7)**	**p**	**T0 PNP (+) (n=4)**	**T1 PNP (+) (n=4)**	**p**
**H&Y**	3 (IQR 1) (7)	4 (IQR 1) (7)	**0.014*** (7)	3 (IQR 1.5) (4)	4 (IQR 2.3) (4)	0.083 (4)
**MDS-UPDRS I**	10.29 ± 5.68 (7)	17.5 ± 7.95 (6)	0.075 (6)	10.5 ± 7.85 (4)	17 ± 11.27 (3)	0.285 (3)
**MDS-UPDRS II**	18.71 ± 10.28 (7)	27.4 ± 17.95 (5)	0.080 (5)	18.25 ± 13.89 (4)	17.33 ± 16.26 (3)	0.285 (3)
**MDS-UPDRS IIII**	40.29 ± 16.09 (7)	46.5 ± 13.8 (6)	0.345 (6)	42.25 ± 18.36 (4)	49 ± 20.88 (3)	1.0 (3)
**PDQ 39**	26.88 ± 18.63 (5)	40.21 ± 21.69 (6)	0.465 (4)	31.67 ± 23.49 (3)	36.11 ± 31.19 (3)	0.180 (2)

**NMSQ**	8.71 ± 4.07 (7)	8 ± 6.16 (6)	0.833 (6)	9 ± 5.66 (4)	5 ± 2.65 (3)	0.285 (3)
**NSS**	1.86 ± 3.76 (7)	3.33 ± 2.84 (6)	0.588 (6)	3.25 ± 4.72 (4)	4 ± 3.16 (4)	0.854 (4)
**NDS**	5.29 ± 0.95 (7)	4.57 ± 3.16 (7)	0.609 (7)	5.25 ± 0.96 (4)	5 ± 4.08 (4)	1.0 (4)
**PSP RS I-VI**	41.67 ± 15.15 (6)	43.4 ± 19.01 (5)	0.141 (4)	42.67 ± 18.36 (3)	42.33 ± 26.63 (3)	0.655 (2)
**PSP RS I**	8.50 ± 4.37 (6)	8.8 ± 5.81 (5)	0.068 (4)	8.33 ± 4.73 (3)	9.33 ± 8.08 (3)	0.180 (2)
**PSP RS II**	6.17 ± 1.17 (6)	4.2 ± 3.11 (5)	0.414 (4)	6.67 ± 1.53 (3)	3.33 ± 3.21 (3)	0.317 (2)
**PSP RS III**	2.33 ± 2.16 (6)	3 ± 1.87 (5)	0.059 (4)	2.33 ± 1.16 (3)	3 ± 1.73 (3)	0.157 (2)
**PSP RS IV**	8.50 ± 2.88 (6)	8 ± 2.55 (5)	0.257 (4)	7.67 ± 4.16 (3)	8 ± 3.46 (3)	0.655 (2)
**PSP RS V**	4.67 ± 1.86 (6)	6.2 ± 2.28 (5)	0.059 (4)	5.33 ± 2.52 (3)	7 ± 2.65 (3)	0.180 (2)
**PSP RS VI**	11.50 ± 4.76 (6)	13.2 ± 7.01 (5)	0.068 (4)	12.33 ± 5.69 (3)	11.67 ± 9.45 (3)	0.180 (2)
**MoCA**	19.71 ± 2.98 (7)	17.17 ± 5.85 (6)	0.246 (6)	21.5 ± 2.52 (4)	21.33 ± 4.51 (3)	1.0 (3)
**LED (mg)**	493. 14 ± 404.22 (7)	622.14 ± 349.53 (7)	0.128 (7)	463 ± 348.92 (4)	570 ± 342.66 (4)	0.144 (4)
**Levodopa (mg)**	357.14 ± 282.00 (7)	414.29 ± 267.26 (7)	0.414 (7)	325 ± 221.74 (4)	325 ± 221.74 (4)	1.0 (4)
**Vitamin B12 (pg/ml)**	496.57 ± 318.23 (7)	367 ± 130.67 (4)	0.273 (4)	357.75 ± 51.86 (4)	400.5 ± 14.85 (2)	0.655 (2)
**Holotranscobalamin (pmol/l)**	95.26 ± 56.80 (7)	51.38 ± 8.92 (4)	0.144 (4)	96.73 ± 67.65 (4)	57.8 ± 0.71 (2)	0.655 (2)
**Folic acid (ng/ml)**	8.37 ± 5.28 (7)	4.14 ± 1.36 (4)	0.068 (4)	6.19 ± 1.37 (4)	4.25 ± 2.35 (2)	0.180 (2)
**Methylmalonic acid (nmol/l)**	232.91 ± 155.49 (7)	266.45 ± 67.96 (4)	0.715 (4)	213.9 ± 136.13 (4)	257.15 ± 16.48 (2)	0.180 (2)
**Homocysteine (μmol/l)**	19.37 ± 6.03 (6)	17.03 ± 5.15 (4)	0.285 (3)	22.5 ± 7. 16 (3)	17.05 ± 7.42 (2)	0.317 (1)
**Sural nerve (μV)**	2.74 ± 1.94 (7)	3.45 ± 5.68 (6)	1.00 (6)	1.6 ± 1.85 (4)	4.83 ± 8.37 (3)	0.317 (3)
**Tibial nerve (mV)**	6.57 ± 2.38 (7)	6.06 ± 2.1 (7)	0.500 (7)	5.75 ± 2.52 (4)	5.45 ± 2.28 (4)	0.593 (4)
**Median sensory nerve (μV)**	5.93 ± 2.65 (7)	5.59 ± 7.5 (7)	0.499 (7)	6.75 ± 3,25 (4)	8.98 ± 8.63 (4)	0.715 (4)
**Median motor nerve**	5.2 ± 1.95 (7)	5.44 ± 3.02 (7)	0.866 (7)	5.43 ± 2.13 (4)	6.6 ± 3.27 (4)	0.465 (4)
**Fibular motor nerve (mV)**	3.23 ± 2.39 (6)	2.74 ± 2.62 (5)	0.465 (4)	2.7 ± 2.79 (3)	3.55 ± 5.02 (2)	0.317 (1)
**Fibular sensory nerve (μV)**	0.57 ± 1.39 (6)	0 ± 0 (5)	0.317 (4)	1.13 ± 1.96 (3)	0 ± 0 (2)	0.317 (1)
**Radial nerve (μV)**	5.43 ± 0.62	5.55 ± 3.3	0.893	5.3 ± 0.46	6.6 ± 0.75	0.180

	(6)	(6)	(5)	(3)	(3)	(2)
**Ulnar motor nerve (mV)**	6.43 ± 2.51 (6)	7.83 ± 2.42 (6)	0.138 (5)	6.33 ± 3.25 (3)	8.5 ± 4.5 (3)	0.180 (2)
**Ulnar sensory nerve (μV)**	4.8 ± 2.64 (6)	3.27 ± 2.41 (6)	0.225 (5)	4 ± 3.9 (3)	4.7 ± 2.19 (3)	0.180 (2)

* Clinical scores: mean values ± SD are presented. H&Y scale: median value and IQR are presented. NCS: mean amplitudes ± SD are presented. H&Y: median and IQR are presented. *p < 0.05, **p < 0.01

Table S5. Differences between bootstrapping and nonparametric tests MSA T0-T2 cohort.

	**T0 total (n=6)**	**T2 total (n=6)**	**n**	**Lower KI**	**Higher KI** (samples)[a]
NDS	2.4 ± 2.3	5 ± 3	5	-4	-1 *(595)*
UMSARS I	13 ± 4.08	21.75 ± 5.74	4	-12	-5 *(893)*

Clinical scores: mean values ± standard deviation (SD) are presented. Confidence intervals were considered significant if they did not include the value zero. [a]if not otherwise stated, bootstrapping was based on 1000 samples.

Table S6. Differences between bootstrapping and nonparametric tests MSA T0-T2 PNP positive cohort

	**T0 PNP + (n=3)**	**T2 PNP + (n=3)**	**n**	**Lower KI**	**Higher KI** (samples)[b]
H&Y	3.33 ± 0.58	3.67 ± 0.58	3	-0.75[a]	-0.2 *(581)*
MDS-UPDRS II	21 ± 5.66	33.5 ± 4.95	2	-12.75[a]	-12.25 *(401)*
NSS	7.5 ± 0.71	6 ± 0	2	1.25[a]	1.75 *(414)*
MoCA	19.33 ±1.53	17.33 ± 2.08	3	0.75[a]	2.4 *(576)*
Vitamin B12 (pg/ml)	311 ± 83.44	414.5 ± 123.74	2	-117.75[a]	-89.25 *(416)*
Sural nerve (μV)	0 ± 0	1.96 ±3.4	3	-4.43[a]	-1.18 *(654)*
Tibial nerve (mV)	4.73 ± 2.02	3.08 ± 2.1	3	1.52[a]	1.79 *(760)*
Median sensory nerve (μV)	8.57 ± 6.82	4.8 ± 1.75	3	0.23[a]	7.07 *(749)*
Median motor nerve (mV)	5.67 ± 3.22	6.43 ± 4.29	3	-1.58[a]	-0.03 *(730)*

Clinical scores: mean values ± standard deviation (SD) are presented. Confidence intervals were considered significant if they did not include the value zero. [a] if it could not be calculated using the BCa method, then the percentile method was used, [b] if not otherwise stated, bootstrapping was based on 1000 samples.

Table S7. Differences between bootstrapping and nonparametric tests PSP T0-T2 cohort

	**T0 total (n=6)**	**T2 total (n=6)**	**n**	**Lower KI**	**Higher KI** *(samples)[b]*
H&Y	3 ± 0.63	3.17 ± 0.75	6	-0.57[a]	-0.11 *(648)*
MDS-UPDRS I	11 ± 6.63	14.33 ± 5.57	6	-6.4	-0.33 *(993)*
NDS	4.5 ± 1.52	5.67 ± 2.58	6	-2.79	-0.17 *(894)*

PSP RS I	5.25 ± 4.57	9 ± 2.71	4	-5	-3 *(942)*
PSP RS V	4.2 ± 1.3	5 ± 2	5	-2	0.2 *(921)*
Levodopa (mg)	133.33 ± 206.56	275 ± 331.29	6	-300	-10 *(967)*
median sensory nerve (μV)	8.18 ± 3.76	5.7 ± 1.55	5	0.65	4.58 *(977)*

Clinical scores: mean values ± standard deviation (SD) are presented. Confidence intervals were considered significant if they did not include the value zero. [a] if it could not be calculated using the BCa method, then the percentile method was used, [b] if not otherwise stated, bootstrapping was based on 1000 samples.

Table S8. Longitudinal evaluation of the MSA cohort at T0, T1 and T2

	**T0**	**T1**	**T2**	**p**
**H&Y (4)**	3 (IQR 0,4)	3 (IQR 1,1)	3,5 (IQR 2,5)	0.717
**MDS-UPDRS I (2)**	6.5 + 0.71	10 + 8.49	12 + 2.83	0.607
**MDS-UPDRS II (2)**	15.5 + 0.71	18 + 5.66	26.5 + 9.19	0.223
**MDS-UPDRS III (3)**	32.67 + 19.22	52.33 + 20.98	43.33 + 9.02	0.178
**PDQ 39 (2)**	8.59 + 9.57	19.17 + 5.52	31.95 + 18.08	0.135
**NMSQ (3)**	5.33 + 2.31	5.33 + 2.08	10 + 2.65	0.097
**NSS (4)**	3.25 + 3.95	5.5 + 3.87	4.5 + 3	0.420
**NDS (4)**	2 + 2.45	3.75 + 2.87	4.75 + 3.4	0.116
**UMSARS I (2)**	11 + 5.66	17.5 + 0.71	20.5 + 7.78	0.223
**UMSARS II (1)**	10	21	17	-
**UMSARS III -**	-	-	-	-
**UMSARS IV (1)**	2.5	2	1	-
**MoCA (4)**	24 + 2.94	22.5 + 2.65	20.75 + 4.43	0.247
**LED (mg) (4)**	532.75 + 225.47	624.58 + 191.05	808.33 + 343.19	0.057
**Levodopa (mg) (3)**	316.67 + 76.38	400 + 0	566.67 + 152.75	0.082
**Vitamin B12 (pg/ml) (3)**	353 + 180.14	469.67 + 265.16	347.67 + 86.19	0.717
**Holotranscobalamin (pmol/l) (3)**	76,33 + 33,49	102.63 + 39.71	77.17 + 8.29	0.264
**Folic acid (ng/ml) (3)**	9,24 + 4,21	11.81 + 7.24	5.79 + 2.08	0.060
**Methylmalonic acid (nmol/l) (2)**	448.7 + 170.55	397.65 + 137.96	583.05 + 51.83	0.607
**Homocysteine (μmol/l) (3)**	17.1 + 2.25	16.3 + 3.4	18.53 + 2.04	0.529
**Sural nerve (μV) (4)**	4.2 + 2.91	2.93 + 3.53	2.93 + 5.85	0.761
**Tibial nerve (mV) (4)**	6.1 + 4.53	4.26 + 5.55	4.14 + 4.53	**0.039***
**Median sensory nerve (μV) (4)**	4.45 + 1.51	4.9 + 2.74	2.95 + 1.84	0.472
**Median motor nerve (4)**	4.88 + 2.21	5.75 + 2.7	5.18 + 2.27	0.247
**Fibular motor nerve (mV) (3)**	4 + 2.69	2.52 + 0.99	1.85 + 0.88	0.097
**Fibular sensory nerve (μV) (3)**	0 + 0	3.27 + 5.66	0.42 + 0.73	0.607
**Radial nerve (μV) (3)**	4.23 + 1.17	4.8 + 5.31	5.67 +2.54	0.717
**Ulnar motor nerve (mV) (4)**	6.43 + 1.15	7.9 + 2.18	8.63 + 1.37	0.174
**Ulnar sensory nerve (μV) (4)**	6.28 + 1.04	4.45 + 3.25	2.1 + 2.84	0.105

*The Friedman test was employed to analyze the data from all three periods. Clinical scores: mean values ± SD are presented. H&Y scale: median value and IQR are presented. NCS: mean amplitudes ± SD are presented. H&Y: median and IQR are presented. *p < 0.05.

Table S9. Longitudinal evaluation of the PSP cohort at T0, T1 and T2

	**T0**	**T1**	**T2**	**p**
**H&Y (4)**	3 (IQR 0,8)	4 (IQR 1,5)	3 (IQR 0,8)	0.050
**MDS-UPDRS I (4)**	12 ± 7.26	16 ± 9.42	14.75 ± 7.14	0.472
**MDS-UPDRS II (3)**	13 ± 11.14	17.33 ± 16.26	20.67 ± 15.01	0.097
**MDS-UPDRS III (3)**	33.67 ± 16.26	41 ± 17.09	34 ± 14.73	0.264
**PDQ 39 (2)**	25.5 ± 29.58	20.81 ± 23.24	24.92 ± 23.83	0.223
**NMSQ (4)**	9 ± 5.66	4.25 ± 2.63	10.25 ± 7.14	0.549
**NSS (3)**	4.33 ± 5.13	3 ± 3	5.67 ± 4.93	0.529
**NDS (4)**	5.25 ± 0.96	3.25 ± 3.20	5.5 ± 0.58	0.368
**PSP RS I - VI (2)**	39 ± 16.97	45.5 ± 4.95	33.5 ± 7.78	0.607
**PSP RS I (2)**	8 ± 5.66	10.5 ± 4.95	10.5 ± 3.54	0.223
**PSP RS II (2)**	6.5 ± 2.12	5 ± 4.24	3 ± 0	0.607
**PSP RS III (2)**	1.5 ± 2.12	2.5 ± 2.12	0.5 ± 0.71	0.156
**PSP RS IV (2)**	8.5 ± 0.71	6.5 ± 0.71	5 ± 0	0.135
**PSP RS V(2)**	4 ± 1.41	6 ± 2.83	4 ± 1.41	0.135
**PSP RS VI (2)**	10.5 ± 4.95	15 ± 0	10.5 ± 2.12	0.223
**MoCA (4)**	21.5 ± 2.52	20 ± 4.55	19.25 ± 3.5	0.282
**LED (mg) (4)**	338 ± 414.62	510 ± 323.45	610 ± 483.17	0.076
**Levodopa (mg) (4)**	200 ± 230.94	300 ± 200	412.5 ± 327.55	0.097
**Vitamin B12 (pg/ml) (3)**	358 ± 70.06	327.33 ± 127.16	324 ± 104.54	0.368
**Holotranscobalamin (pmol/l) (3)**	98.03 ± 85.09	51.5 ± 10.92	55.67 ± 9.52	0.717
**Folic acid (ng/ml) (3)**	6.27 ± 0.29	4.19 ± 1.66	7.86 ± 5.67	0.264
**Methylmalonic acid (nmol/l) (3)**	247.23 ± 235.31	290.47 ± 58.87	226.83 ± 33.07	0.717
**Homocysteine (µmol/l) (2)**	16.8 ± 2.4	12.65 ± 1.2	14.95 ± 2.47	0.135
**Sural nerve (µV) (4)**	1.83 ± 2.13	3.63 ± 7.25	2.98 ± 3.9	1.0
**Tibial nerve (mV) (4)**	6.95 ± 2.42	6.78 ± 2.46	6.59 ± 4.06	0.936
**Median sensory nerve (µV) (4)**	6.95 ± 2.96	9.25 ± 8.35	5.3 ± 1.46	0.779
**Median motor nerve (4)**	5.4 ± 2.16	5.28 ± 3.4	4.7 ± 1.52	0.779
**Fibular motor nerve (mV) (2)**	4.05 ± 2.33	4.65 ± 3.46	5.45 ± 0.92	1.0
**Fibular sensory nerve (µV) (2)**	1.7 ± 2.4	0 ± 0	0 ± 0	0.368
**Radial nerve (µV) (1)**	5.8	6.5	9.8	-
**Ulnar motor nerve (mV) (1)**	7	11.1	8	-
**Ulnar sensory nerve (µV) (1)**	4.2	6.3	3.8	-

*The Friedman test was employed to analyze the data from all three periods. Clinical scores: mean values ± SD are presented. H&Y scale: median value and IQR are presented. NCS: mean amplitudes ± SD are presented. H&Y: median and IQR are presented. *p < 0.05.

Figure S1. (a) Distribution of PNP severity levels in MSA and PSP patients: a) at baseline; b) at one.-year follow-up (T1)

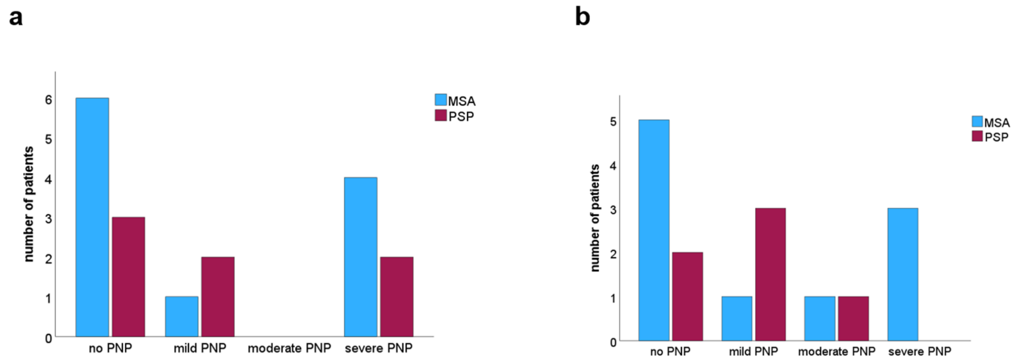

Figure S2. (a) Distribution of PNP severity levels in MSA and PSP patients: a) at baseline; b) at one-year follow-up (T1)

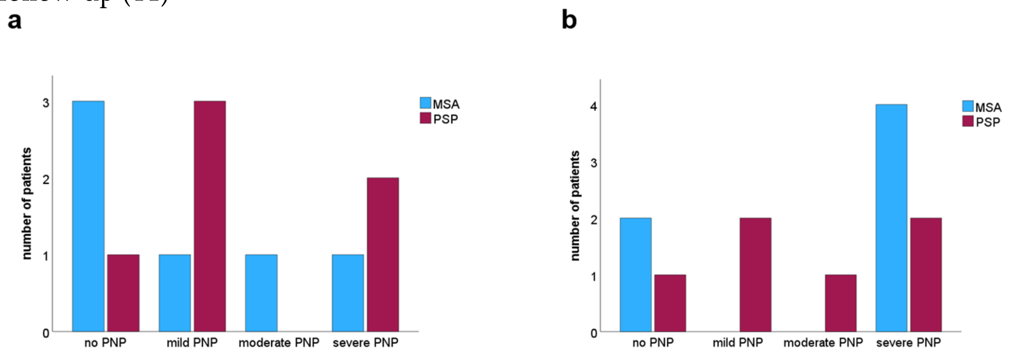

Figure S3. (a) Distribution of PNP severity levels of MSA and PSP patients: a) at baseline; b) at one-year follow-up (T1); c) at two year follow-up (T2)

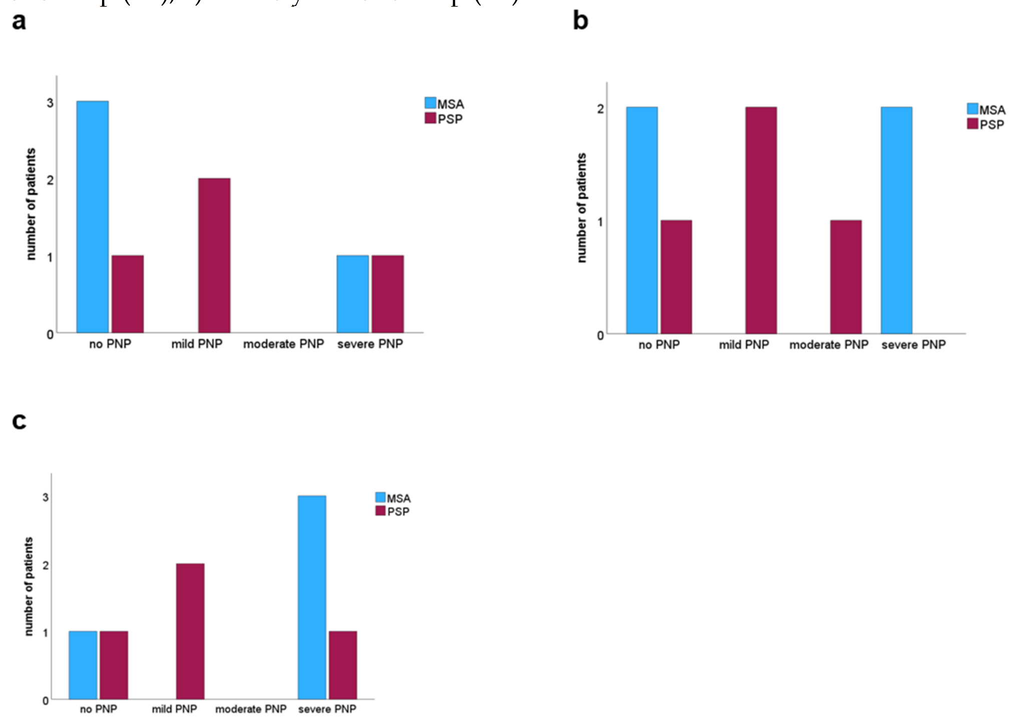

Supplement: Supplementary file 1 [file neurolint-18-00027-s001.zip › neurolint-4046730-supplementary.pdf]
